# Exploratory Analysis of Behavioral Impulsivity, Pro-inflammatory Cytokines, and Resting-State Frontal EEG Activity Associated With Non-suicidal Self-Injury in Patients With Mood Disorder

**DOI:** 10.3389/fpsyt.2020.00124

**Published:** 2020-02-26

**Authors:** Ji Sun Kim, Eun-Sook Kang, Yong Chun Bahk, Sunglee Jang, Kyung Sue Hong, Ji Hyun Baek

**Affiliations:** ^1^Department of Psychiatry, Sooncheonhyang University Cheonan Hospital, Cheonan, South Korea; ^2^Department of Laboratory Medicine, Samsung Medical Center, Sungkyunkwan University School of Medicine, Seoul, South Korea; ^3^Samsung Biomedical Research Institute, Seoul, South Korea; ^4^Department of Psychiatry, Samsung Medical Center, Sungkyunkwan University School of Medicine, Seoul, South Korea

**Keywords:** non-suicidal self-injury, behavioral impulsivity, mood disorder, TNF-α, resting Qeeg, frontal theta

## Abstract

**Introduction:** Non-suicidal self-injury (NSSI) is a rapidly increasing mental health problem that requires more clinical attention. In this study, we aimed to explore the biobehavioral markers of NSSI in participants with mood disorders.

**Methods:** A total of 45 participants with mood disorders (bipolar I, II, and major depressive disorder) were included in the study. Behavioral impulsivity was measured using the immediate memory task (IMT)/delayed memory task (DMT) and the go-no-go (GNG) tests. Plasma levels of tumor necrosis factor-α (TNF-α), interleukin 1 beta (IL-1 β), and interleukin 6 (IL-6) and resting-state quantitative electroencephalography (qEEG) were measured.

**Results:** The NSSI group had shorter GNG reaction time (GNG-RT) and higher TNF-α levels compared to the non-NSSI group. TNF-α was positively correlated with frontal theta power. In addition, GNG-RT showed a significant positive association with frontal alpha activity.

**Conclusion:** NSSI in mood disorders was associated with increased behavioral impulsivity and greater inflammation. Increased pro-inflammatory cytokines were associated with frontal theta power. Increased inflammation might change major neurotransmitter metabolism, which eventually affects frontal function and decreases response inhibition. Further studies to explore their causal relationship are warranted.

## Introduction

Non-suicidal self-injury (NSSI), defined as the direct and deliberate destruction of one's own bodily tissue in the absence of suicidal intent, has recently increased among young people (1). NSSI can be distressing, not only to the person who injures but also to their family and friends. Furthermore, NSSI is the strongest predictor for future suicidal behavior (2). However, little is known about its neurobiological mechanisms and biomarkers.

One mechanism that can link NSSI to suicide is impulsivity. In general, previous studies reported that NSSI was associated with increased impulsivity (3, 4). However, it is still unknown what types of impulsivity is directly associated with NSSI. Impulsivity is a multidimensional construct that embraces both traits and states. A recent meta-analysis of task-based measures of impulsivity suggested that behavioral impulsivity, difficulty preventing the initiation of a behavior or stopping a behavior that has already been initiated (5), could be a proximal risk factor for NSSI (4).

Although limited studies exist on the biomarkers associated with NSSI, there are indirect evidences that has suggested the potential biomarkers. Inflammatory markers were shown to be one of the key markers associated with suicide and other impulsive behavioral problems. Previous studies showed that suicide was associated with inflammation, even after adjusting for depression severity (6). Moreover, several studies have reported the association between impulsivity and inflammation (7, 8).

From a neuroanatomical perspective, impulse control is associated with prefrontal functioning. Several studies have shown that frontal dysfunction observed in the resting frontal cortical activity measured using quantitative electroencephalography (qEEG) was associated with increased impulsivity (9–11). Therefore, impulsive behavior, including NSSI, might be associated with frontal dysfunction.

In this study, we aimed to explore the biomarkers of NSSI in patients with mood disorders, focusing on behavioral impulsivity, proinflammatory cytokines, and resting frontal qEEG activity.

## Materials and Methods

### Participants

Participants who met the diagnostic criteria for bipolar I (BP-I), II (BP-II), and major depressive disorder were recruited from the outpatient and inpatient units of the Samsung Medical Center between January 2017 and December 2018. The participants ranged in age from 18 to 45 years and were clinically stable, i.e., scored 3 (mildly ill) or lower on the Clinical Global Impression of Severity scale (12) at the time of assessment. We excluded those who had evidence of schizophrenia or schizoaffective disorders, organic mental disorder, intellectual disability, or medical illness related to mental symptoms.

A total of 45 participants were included in the analysis. Written informed consent was obtained from all participants. This study was approved by the Institutional Review Board of the Samsung Medical Center (IRB no. 2016-11-081).

### Assessment

Information was collected through direct interviews with the patients, their available caregivers, and their physicians. Patient medical records were also used as a supplementary information source. A board-certified psychologist conducted the direct interviews and medical record reviews.

The participants' diagnoses were confirmed using a Mini-international Neuropsychiatric Interview (MINI). Suicide history was also confirmed using the MINI suicide module.

### History of NSSI

History of NSSI was determined using the information collected through the direct interviews and medical record reviews. A board-certified psychologist who was blinded to the subject's clinical course double-checked all available information and confirmed a history of NSSI. The participants were classified into two groups based on a history of NSSI, the NSSI group and the non-NSSI group. The NSSI group in our study was comprised of patients who consistently reported engaging in NSSI to our research team, their clinicians, and family members. The non-NSSI group included those who consistently denied the experience of NSSI to our research team, their clinicians, and family member.

### Mood Symptom Evaluation

The participants' current mood states were measured using the Young Mania Rating Scale (YMRS), the Hamilton Depression Rating Scale (HAM-D) (13) and the Hamilton Anxiety Rating Scale (HAM-A).

### Impulsivity

Trait impulsivity was measured using the Barratt impulsivity scale (BIS-11) (14). The BIS-11 is a widely used self-report questionnaire designed to assess the personality/behavioral construct of impulsivity. It has 30 items with 4-point scale.

Behavioral impulsivity was measured using the cued go-no-go task (GNG) and the immediate memory task (IMT)/delayed memory task (DMT) recommended by the International Society for Research on Impulsivity. The cued GNG (15) measures impulse control by the ability to inhibit instigated prepotent responses. The IMT/DMT (16) is a continuous performance test that was designed to assess impulsive behavior. The IMT involves comparing consecutively presented numbers and responding when the current number matches the number immediately before it (called a correct detection). The DMT similarly involves responding to matching numbers but the numbers to be compared are separated by a filler sequence (e.g., 12345). Commission error (%) and response time (msec) were utilized to measure behavioral impulsivity.

### Pro-inflammatory Cytokines

EDTA-anticoagulated whole blood was obtained from the patients between 9 and 11 a.m. by forearm venipuncture. Specimens were centrifuged at 3,500 rpm for 10 min. Plasmas were stored at −70°C within 4 h after collection. Interleukin(IL)-1, IL-6 and tumor necrosis factor alpha (TNF-α) were measured using a multiplexed electrochemiluminescence based assay (V-plex Customized proinflammatory panel, Meso Scale Discovery, Rockville, MD, USA). All assays were performed in duplicate with the average recorded. The lower limit of detection of IL-1, IL-6, and TNF-α were 0.01, 0.05, and 0.01 pg/mL, respectively.

### Resting qEEG Measures

EEGs were recorded using a NeuroScan SynAmps 2 amplifier (Compumedics, El Paso, TX, USA) from 21 surface electrodes mounted on a Quik-Cap (Compumedics, El Paso, TX, USA) using the extended international 10−20 placement scheme. The participants were seated in a dimly lit, sound-attenuated room. They were asked to relax and remain still for 5 min with their eyes closed. The ground electrode was placed on the forehead, while the reference electrode was predefined in the cap and positioned between Cz and CPz. The impedance of the electrode was maintained at <5 kΩ.

The EEG data were recorded with a 0.1–100 Hz band-pass filter at a sampling rate of 1,000 Hz, initially processed using Scan 4.3 (17). Eye movements were visually screened and eliminated by an expert. The recorded EEG data were preprocessed using CURRY 8. Gross artifacts were rejected through visual inspection by a trained person with no prior information regarding the origin of the data. Artifacts related to eye movement or eye blinks were removed using a mathematical procedure implemented in the pre-processing software (18). The signal was segmented using predefined time windows of 2.048 s each. Epoch with signals over +80 μV or lower than −80 μV on any channel was removed from the analysis. A total of 30 epochs (~60) were prepared for each subject. Spectral density was calculated in each epoch on 23-electrode channels and averaged 30 epochs by Fast Fourier Transform (FFT). The accepted epochs of the EEG data for both absolute (uV2) and relative (%) power were smoothed using fast Fourier transforms and averaged in four frequency bands by NeuroGuide's spectral analysis system. After performing FFT, the spectral density was averaged into specific frequency ranges as follows: delta (1–4 Hz), theta (4–8 Hz), alpha (8–12 Hz), beta (12–30 Hz), and low gamma (30–50 Hz) (19, 20). The relative power of each channel was calculated by dividing each band power by the total power of the channel. Since abnormalities in resting qEEG associated with impulsivity were commonly detected in the frontal region (9), we focused on the frontal region when examining the resting qEEG measures. The absolute and relative EEG power within each frequency band from the individual scalp electrodes was averaged across the frontal region.

### Statistical Analyses

We conducted chi-squared tests to compare the rates of categorical variables and Students' *t*-tests to compare the mean values of continuous variables between the NSSI and the non-NSSI groups. The levels of pro-inflammatory cytokines and frontal qEEG measures were natural-log transformed (ln) to normalize the data. We performed analyses of covariance (ANCOVA) to compare impulsivity and the pro-inflammatory cytokine levels between the two groups. Age and sex were entered as covariates for impulsivity measures, and age, sex and body mass index (BMI) were entered as covariates for the pro-inflammatory cytokine levels. Partial correlation analysis was performed to evaluate the relationship between frontal qEEG measures and other variables after adjusting for age, sex, and BMI. We additionally conducted linear regression analysis for frontal qEEG measures and neurobehavioral markers for NSSI that showed significant associations in the partial correlation analyses. The significance level was set at *p* < 0.05. All statistical analyses were performed using SPSS software (IBM SPSS Statistics for Windows, version 24.0; IBM Inc. Armonk, NY, USA).

## Results

Of the 45 participants included in the analysis, 23 were classified into the NSSI group. No significant difference was observed in terms of age, sex, and diagnosis between the two groups (Table 1). No significant difference was observed in terms of severity of mood symptoms between the two groups. Patients in the NSSI group were younger age at onset than the non-NSSI group. All participants took psychotropic medication (Table 2).

**Table 1:** Sociodemographic characteristics and basic symptom characteristics of participants.

	**NSSI group (*n* = 23)**	**Non-NSSI group (*n* = 22)**	***t*, *X*^**2**^**	***p***
Age [years, mean (SD)]	24.09 (5.09)	27.05 (7.03)	−1.62	0.112
Sex [males, *n* (%)]	9 (39.1)	10 (45.5)	0.18	0.668
Handedness [right, *n* (%)]	21 (91.4)	21 (95.5)	0.31	0.577
Diagnosis			2.04	0.361
BP-I	5 (21.7)	9 (40.9)		
BP-II	10 (43.5)	8 (36.4)		
MDD	8 (34.8)	5 (22.7)		
BMI	24.32 (4.22)	24.14 (4.72)	0.13	0.901
YMRS	2.61 (2.19)	3.55 (4.89)	−0.82	0.417
HAMD	11.22 (4.09)	11.91 (7.46)	−0.38	0.704
HAMA	11.17 (3.89)	11.36 (7.95)	−1.35	0.183
History of suicide attempt [present, *n* (%)]	11 (47.8)	7 (31.8)	1.20	0.273
Age at onset	16.96 (3.78)	20.96 (6.21)	−2.62	0.012
Duration of education [years, mean (SD)]	13.78 (1.51)	14.14 (1.70)	−0.740	0.463
Duration of illness [month, mean (SD)]	74.61 (52.88)	83.18 (66.32)	−0.48	0.633

*BP-I, bipolar I disorder; BP-II, bipolar II disorder; MDD, major depressive disorder; NSSI, non-suicidal self-injury*.

**Table 2:** Medication currently used for participants.

	**NSSI group (*n* = 23)**	**Non-NSSI group (*n* = 22)**	***X^**2**^***	***p***
On mood stabilizer [*n* (%)]	15 (68.2)	17 (73.9)	0.18	0.672
On atypical antipsychotics [*n* (%)]	19 (86.4)	18 (78.3)	0.51	0.477
On antidepressant [*n* (%)]	10 (45.5)	6 (26.1)	1.84	0.175
On benzodiazepine [*n* (%)]	16 (72.7)	10 (43.5)	3.94	0.047

*NSSI, non-suicidal self-injury*.

**Figure 1:**
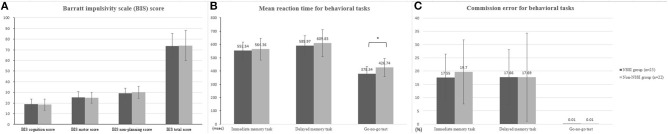
Comparison of behavioral impulsivity between NSSI and non-NSSI group. **(A)** Comparison of Barratt impulsivity (BIS) score **(B)** mean reaction time for behavioral tasks **(C)** commission error for behavioral tasks. NSSI, non-suicidal self-injury. Covariates: age and sex. *p < 0.05.

**Figure 2:**
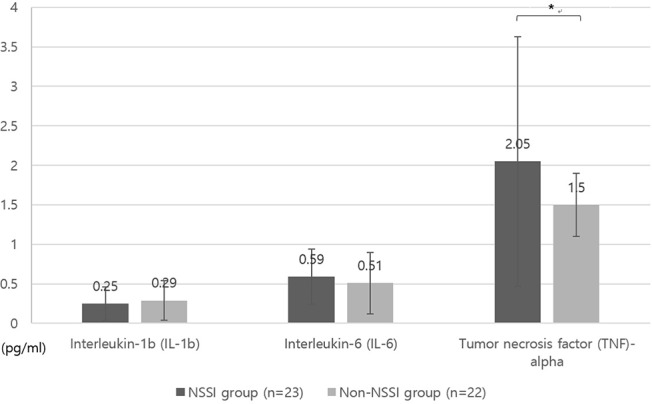
Comparison of levels of inflammatory markers between NSSI and non-NSSI groups. NSSI, non-suicidal self-injury. Covariates: age, sex, and body mass index (BMI). *p < 0.05.

Figure 1 displays the differences in impulsivity between the two groups. No significant difference was observed in terms of the BIS-11 scores (Figure 1A). The NSSI group showed shorter GNG reaction times (GNG-RT) compared to the non-NSSI group after adjusting for age and sex (*F* = 4.49, *P* = 0.04, η^2^ = 0.099; Figure 1B). No significant difference was observed in commission error of GNG, IMT, and GMT (Figure 1C). In addition, the NSSI group had higher TNF-α levels than the non-NSSI group after adjusting for age, sex, and BMI (*F* = 5.59, *P* = 0.025, η^2^ = 0.161; Figure 2).

**Figure 3:**
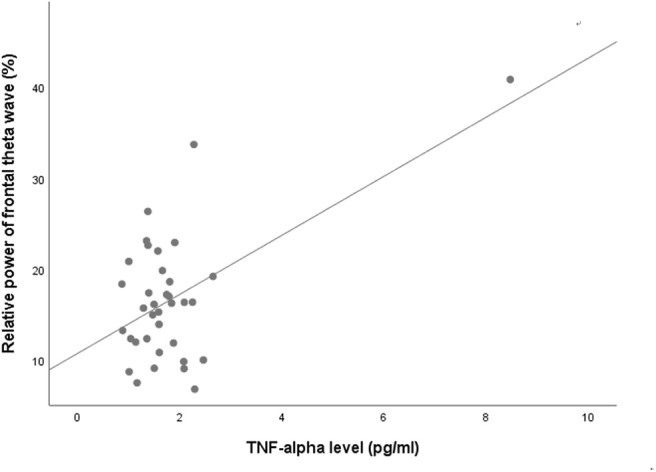
Scatterplot for the relationship between TNF-alpha level and relative power of frontal theta wave.

**Figure 4:**
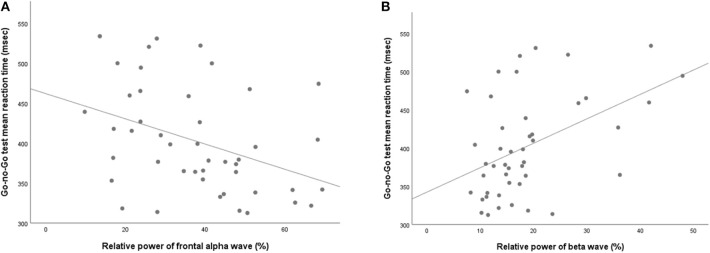
Scatterplot for the relationship between Go-no-Go test mean reaction time and relative powers of **(A)** alpha wave and **(B)** beta wave.

NSSI did not show any significant association with either absolute or relative powers of frontal qEEG measures (Table 3). In exploring the association between impulsivity and pro-inflammatory cytokines, TNF-α showed a positive correlation with the relative power of the frontal theta waves after adjusting for age, sex and BMI (rho = 0.422, *p* = 0.018). Linear regression analysis as relative power of frontal theta wave as a dependent variable showed significant association with TNF-α after adjusting for age, sex and BMI (*R*^2^ = 0.340, *F* = 3.74, *P* = 0.014, standardized β = 0.593, *t* = 3.59, *P* = 0.001; Figure 3). Of the measures of behavioral impulsivity, GNG-RTs showed a negative correlation with frontal alpha power (rho = −0.512, *p* = 0.003) and a positive correlation with frontal beta power (rho = 0.446, *p* = 0.012). Linear regression analysis as GNG-RT as a dependent variable also showed significant associations with frontal alpha power (*R*^2^ = 0.270, *F* = 3.43, *P* = 0.018, standardized β = 0.361, *t* = 2.432, *p* = 0.020; Figure 4A) and frontal beta power (*R*^2^ = 0.266, *F* = 3.36, *P* = 0.019, standardized β = 0.354, *t* = 2.387, *p* = 0.022; Figure 4B). In analyses with absolute power of frontal qEEG measures, Absolute power of frontal beta wave showed significant associations with Go-no-G test reaction time and immediate memory test reaction time (rho = 0.346, *p* = 0.031; rho = 0.414, *p* = 0.009); absolute power of frontal gamma wave showed significant associations with immediate memory test reaction time (rho = 0.376, *p* = 0.018).

**Table 3:** Differences in qEEG parameters between NSSI and non-NSSI group.

	**NSSI group (*n* = 23)**	**Non-NSSI group (*n* = 22)**	***F***	***P***
**Absolute power (μV**^**2**^**)**				
Alpha wave	15.78 (11.97)	15.34 (16.01)	0.04	0.842
Beta wave	6.62 (5.24)	6.77 (4.44)	0.02	0.893
Theta wave	5.51 (2.93)	5.47 (3.17)	<0.01	0.995
Delta wave	6.15 (2.14)	7.12 (2.99)	3.12	0.085
Gamma wave	0.95 (0.82)	1.25 (1.06)	1.22	0.275
**Relative power (%)**				
Alpha wave	39.18 (16.68)	36.77 (15.80)	0.04	0.838
Beta wave	18.37 (8.87)	18.89 (10.11)	0.06	0.810
Theta wave	16.92 (7.88)	15.13 (5.23)	1.43	0.238
Delta wave	19.33 (8.10)	21.00 (8.32)	0.91	0.346
Gamma wave	2.84 (1.86)	3.52 (2.35)	1.03	0.317

*Covariates: age and sex*.

*NSSI, non-suicidal self-injury*.

## Discussion

NSSI is recently increasing among patients with mood disorders. In addition to its association with suicide, NSSI is a highly recurrent and distressing problem that requires more clinical attention. Nonetheless, limited studies have attempted to explore the biobehavioral markers. The present study showed that NSSI was related to greater behavioral impulsivity and increased serum TNF-α level. Additionally, TNF-α showed significant associations with frontal theta power, indicating the association between inflammation and frontal dysfunction. Frontal dysfunction associated with increased behavioral impulsivity and greater inflammation might explain the neurobiological basis of NSSI.

About half of our participants (51.1%) had engaged in NSSI. Previous studies consistently showed that NSSI was highly prevalent in patients with mood disorders (21–23). The presence of mood disorders increased suicide risk in participants engaged in NSSI, suggesting the importance of NSSI in the suicide risk evaluation of patients with mood disorders (2, 24).

Previous studies also showed that early-onset mood disorders were associated with NSSI (25), consistent with our study findings. Early-onset mood disorders also had more severe symptoms and poorer clinical outcomes, which were eventually linked to increased suicide risk (26–28).

Systematic reviews generally support an association between impulsivity and NSSI. However, we cannot directly apply the study findings to precise risk evaluations for NSSI. This could be related to variations in the concept and assessment of impulsivity across the studies. Most studies used self-reported questionnaires to measure impulsivity. Aside from the various limitations of self-reported measures, including recall bias, impression management, and motivation, in evaluating impulsivity (29), self-reported measures mainly examine trait impulsivity. Although notable as an indicator of general risk, trait impulsivity, a fixed marker of risk by definition, cannot inform the causal risk factors for NSSI (30). A previous meta-analysis (4) suggested behavioral impulsivity as an imminent risk for NSSI. Another review study pointed out that mood-based impulsivity, impulsivity associated with mood change, was related to the initiation of self-harm, while the cognitive aspects of impulsivity were associated with the maintenance of self-harm (31). Considering that mood-based impulsivity is generally increased in participants with mood disorders compared to the general population, participants with mood disorders who have greater behavioral impulsivity could represent a high-risk group for NSSI. Therefore, GNG could be useful to evaluate the imminent risk for NSSI in participants with mood disorders. In our study, the NSSI group had shorter GNG-RTs, i.e., difficulty in response inhibition, compared to the non-NSSI group. Previous studies have reported mixed results regarding the association between behavioral impulsivity and NSSI. Fikke and colleagues (32) reported that impaired inhibitory control was evident in participants who engaged in more severe NSSI. However, other studies have reported negative findings (33, 34). A previous meta-analysis study explained that the negative association could be due to studies that included participants who were not at active risk to engage in NSSI (4). The NSSI group in our study was carefully selected after reviewing all medical records and directly interviewing the participants, their family members, and their treating clinicians. Although we did not explore the frequency and recency of NSSI, all participants in the NSSI group consistently reported the experience of NSSI and were considered to have a high-risk for engaging in NSSI in the near future.

Previous studies mostly focused on the relationships between inflammation and suicide (35). Nevertheless, limited evidence exists on a relationship between inflammation and NSSI. This is the first study to show an association between TNF-α and NSSI. A recent study using Danish national registry data showed that individuals with infections treated with anti-infective agents had an increased risk of NSSI, suggesting the importance of inflammation in the pathophysiology of NSSI (36). Likewise, inflammation showed clear associations with aggressive behavior and impulsivity (7). These findings indicate that the role of central neurotransmitters on aggressive and destructive behaviors, including NSSI, might be moderated by inflammation.

NSSI generally happens repetitively and habitually. Participants who engage in NSSI often report difficulties in controlling unwanted urges. In that sense, NSSI has many behavioral and cognitive similarities with obsessive-compulsive disorder (OCD) (37). In a mouse model, elevated TNF-α signaling led to OCD-like behavior (38). In this regard, the increased TNF- α observed in our study could be also related to the compulsive nature of self-harming behaviors. In addition, frontal EEG alpha activity showed a significant association with frontal alpha activity. In a previous study of non-clinical young adults, frontal alpha activity was also related to OCD-like behaviors (39). Therefore, we could speculate that behavioral impulsivity reflected by shorter GNG-RT might affect the repetition of self-harm moderated by inflammation, which is also connected to the changes in frontal activity.

Inflammatory cytokines may contribute to activation of the kynurenine pathway of tryptophan catabolism, dysregulation of the hypothalamic-pituitary-adrenal (HPA) axis, and alterations in monoamine metabolism (6). Activation of the kynurenine pathway could result in aberrant glutamate signaling via N-methyl-D-aspartate (NMDA) agonistic activity and inhibit the astrocytic uptake of glutamate. Furthermore, dysregulation of the HPA axis and alteration of monoamine metabolism could alter serotonin metabolism. In addition, increased TNF-α has been associated with chronic microglial activation and white matter degeneration.

Alterations of in the CNS induced by inflammation could cause frontal lobe dysfunction, which could be associated with increased impulsivity (40–42) and compulsive behaviors (43, 44). Previous studies supported an association between frontal lobe dysfunction and NSSI. A recent study by Dahlgren and colleagues showed that women who engaged in NSSI had increased cingulate cortex and decreased dorsolateral prefrontal cortex activation during cognitive tasks (45). Other studies have also suggested changes in the activation pattern within the frontal circuitry related to emotional processing tasks in participants with NSSI (46–48). Decreased volume or gray matter thickness of the frontal region was significantly associated with suicidal behavior in participants with mood disorders (49–53). Likewise, activation of the ventrolateral prefrontal cortex, particularly the right inferior frontal gyrus (rIFG), has been implicated in behavioral impulsivity (40–42). The frontal lobe may inhibit the limbic system, which is probably dysregulated in emotionally unstable individuals and thus, could cause impulsive behavior (50). Hence, NSSI associated with increased behavioral impulsivity might be mediated by frontal dysfunction.

This study did not find direct associations between frontal qEEG measures and NSSI but the findings did support an association between frontal lobe dysfunction, impulsivity, and inflammation in NSSI. Increased frontal theta power, which has been suggested as a biomarker for suicidal ideation (54), showed significant association with TNF-α in our study. Previous studies have collectively suggested that increased inflammation in the frontal region could be associated with an increased risk for NSSI or suicidal behavior. In a study of participants with traumatic brain injury, increased TNF-α in the CNS increased disinhibition (disinhibited behavioral state) (55). Likewise, increased inflammation in the frontal lobe was detected in a postmortem study of suicide victims (56). The GNG RT showed a significant association with frontal alpha activity in our study and the frontal alpha activity might be associated with obsessive-compulsive behaviors (39, 57).

## Limitation

The study findings need to be interpreted in the context of the study design. First, we did not apply DSM-5-defined NSSI to identify the NSSI group in our study. Second, we did not thoroughly explore how often and when the participants engaged in NSSI. Third, the small sample size and multiple comparisons could bias the study results. Lastly, we did not measure qEEG while the participants conducted the tasks used to evaluate behavioral impulsivity.

## Conclusions

We sought to objectively examine biobehavioral markers associated with NSSI. NSSI showed significant associations with behavioral impulsivity and greater inflammation. Inflammation showed significant associations with frontal dysfunction. Increased inflammation might change major neurotransmitter metabolism, which eventually affects frontal function and decreases response inhibition. Further study to confirm the study findings are warranted for clinical application.

## Data Availability Statement

The datasets generated for this study are available on request to the corresponding author.

## Ethics Statement

This study was approved by the Institutional Review Board of the Samsung Medical Center. Written informed consent was obtained from all participants.

## Author Contributions

JB completed initial study design. SJ conducted the experiment. E-SK conducted cytokine analysis and wrote the cytokine analysis section of the manuscript. YB and JB analyzed the data. JK and JB wrote the original manuscript. KH reviewed and edited the original manuscript. All authors finalized submitted version of the manuscript.

### Conflict of Interest

The authors declare that the research was conducted in the absence of any commercial or financial relationships that could be construed as a potential conflict of interest.
